# Non-contiguous finished genome sequence and description of *Halopiger goleamassiliensis* sp. nov.

**DOI:** 10.4056/sigs.4618288

**Published:** 2013-12-31

**Authors:** Hassani Imene Ikram, Robert Catherine, Michelle Caroline, Raoult Didier, Hacène Hocine, Desnues Christelle

**Affiliations:** 1 USTHB Université, Laboratoire de Biologie Cellulaire et Moléculaire, Faculté de Biologie Algérie; 2 Aix-Marseille Université, URMITE, UM63, CNRS 7278,

**Keywords:** *Halopiger goleamassiliensis*, Draft genome, *Euryarchaeota*, Extreme halophile, thermotolerant

## Abstract

*Halopiger goleamassiliensis* strain IIH3^T^ sp. nov. is a novel, extremely halophilic archaeon within the genus *Halopiger*. This strain was isolated from an evaporitic sediment in El Golea Lake, Ghardaïa region (Algeria). The type strain is strain IIH3^T^. *H. goleamassiliensis* is moderately thermophilic, neutrophilic, non-motile and coccus-shaped. Here we describe the features of this organism, together with the complete genome sequence and annotation. The 3,906,923 bp long genome contains 3,854 protein-encoding genes and 49 RNA genes (1 gene is 16S rRNA, 1 gene is 23S rRNA, 3 genes are 5S rRNA, and 44 are tRNA genes).

## Introduction

*Halopiger goleamassiliensis* sp. nov. strain IIH3^T^ (=KC 430940 =CSUR P3036 = DSM on-going deposit) is the type strain of *H .goleamassiliensis* sp. nov. This organism is a Gram-negative, extremely halophilic, moderately thermophilic and strictly aerobic archaeon. It was isolated from evaporitic sediment in El Golea Lake, Ghardaïa region (Algeria) as part of a project studying archaeal diversity in hypersaline Lakes of Algeria.

The number of genera and species belonging to *Halobacteria* (*Archaea*, *Euryarchaeota*) has increased recently due to studies of several different hypersaline environments (thalassohaline and athalassohaline) combined with the use of different isolation media and culture conditions [1]. At the time of writing, the family *Halobacteriaceae*, the single family described within the order *Halobacteriales*, accommodated 40 recognized genera [2]. The genus *Halopiger* was proposed by Gutiérrez et *al*. (2007) [3] and contains only three species, *Halopiger xanaduensis* isolated from the Shangmatala Lake (China) [3], *Halopiger aswanensis* isolated from a hypersaline soil in Aswan (Egypt) [4] and *Halopiger salifodinae* recently isolated from a salt mine in Kuche county, Xinjiang province, China [5]. So far, this genus is composed of strictly aerobic, Gram-negative, polymorphic and pigmented strains. We have recently used [6-18] a polyphasic approach for prokaryotic classification [19] that includes genomic data [20,21], MALDI-TOF spectra [22,23] and major phenotypic characteristics.

Using this approach, we report here a summary classification and a set of features for *Halopiger goleamassiliensis* sp.nov. strain IIH3^T^ together with the description of the complete genomic sequencing and annotation. These characteristics support the circumscription of the *H. goleamassiliensis* species.

### Classification and features

*H. goleamassiliensis* was isolated from an evaporitic sediment of the hypersaline Lake El Golea in Ghardaïa region of Algeria. The sediment sample (1g) was enriched in a liquid SG medium [24] containing ampicillin (100 μg/mL) at 55°C on a rotary shaking platform (150 rpm) for 7 to 15 days. Serial dilutions of enrichment cultures were plated on SG agar plates and incubated aerobically at 55°C. After 2 to 6 weeks of incubation, representative colonies were picked and maintained in the SG medium at 55°C. Strain IIH3^T^ (Table 1) was isolated in 2012 by cultivation in aerobic conditions at 55°C and stored at –80 ºC with 25% (v/v) glycerol.

**Table 1:** Classification and general features of *Halopiger goleamassiliensis* according to the MIGS recommendations [25].

**MIGS ID**	**Property**	**Term**	**Evidence code ^a^**
		Domain *Archaea*	TAS [26]
		Phylum *Euryarchaeota*	TAS [27]
		Class *Halobacteria*	TAS [28,29]
	Current classification	Order *Halobacteriales*	TAS [30-32]
		Family *Halobacteriaceae*	TAS [33,34]
		Genus *Halopiger*	TAS [3]
		Species *Halopiger goleamassiliensis*	IDA
		Type strain IIH3^T^	IDA
	Gram stain	Negative	IDA
	Cell shape	Coccus	IDA
	Motility	Non-motile	IDA
	Sporulation	None	IDA
	Temperature range	Thermophile, between 40°C and 60°C	IDA
	Optimum temperature	55°C	IDA
MIGS-6.3	Salinity	Halophile, 22.5%-25% (optimum)	IDA
MIGS-22	Oxygen requirement	Aerobic	IDA
	Carbon source	Sugar or amino acids	IDA
	Energy metabolism	Heterotrophic	IDA
MIGS-6	Habitat	Salt Lake sediment	IDA
MIGS-15	Biotopic relationship	Free living	IDA
MIGS-14	Pathogenicity	Non-pathogenic	NAS
	Biosafety level	1	NAS
	Isolation	Sediment of El Golea Lake	IDA
MIGS-4	Geographic location	Algeria	IDA
MIGS-5	Isolation time	2012	IDA
MIGS-4.1	Latitude	30-34 N	IDA
MIGS-4.2	Longitude	002-52 E	IDA
MIGS-4.3	Depth	Surface	IDA
MIGS-4.4	Altitude	397	IDA

a) Evidence codes – IDA: Inferred from Direct Assay; TAS: Traceable Author Statement (i.e., a direct report exists in the literature); NAS: Non-traceable Author Statement (i.e., not directly observed for the living, isolated sample, but based on a generally accepted property for the species, or anecdotal evidence). These evidence codes are from the Gene Ontology project [35]. If the evidence is IDA, then the property was directly observed for a live isolate by one of the authors or an expert mentioned in the acknowledgements.

**Figure 1:**
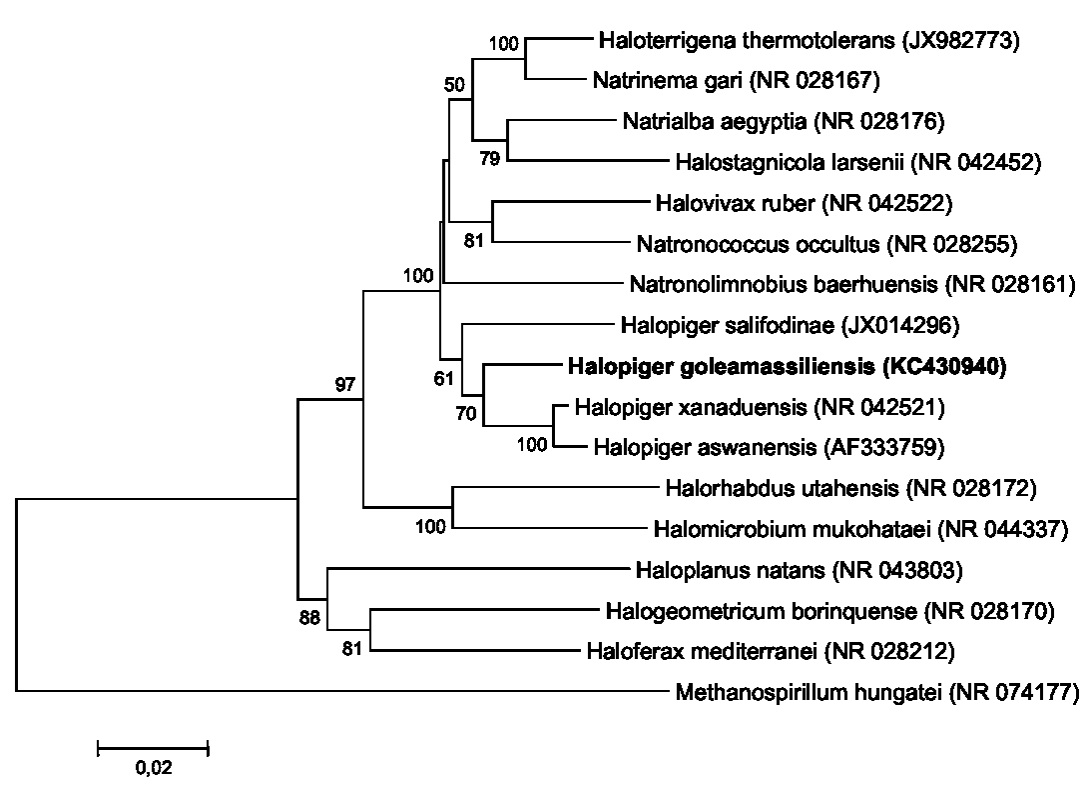
Neighbor-joining phylogenetic tree based on 16S rRNA gene sequence comparisons, showing the position of strain IIH3^T^ and other related haloarchaeal species. GenBank accession numbers are indicated in parentheses. Sequences were aligned using MUSCLE, and phylogenetic inferences obtained using the MEGA software. Numbers at the nodes are from a bootstrap analysis done using 1,000 replicates to generate a majority consensus tree. *Methanospirillum hungatei* was used as outgroup.

Genomic DNA was extracted and purified using the Genomic DNA purification kit (MACHEREY-NAGEL) Hoerd, France. The 16S rRNA gene was amplified by PCR using the primers 21AF: TTCCGGTTGATCCTGCCGGA and RP2: ACGGCTACCTTGTTACGACTT. A total of 1,444 bases were identified. The sequence was compared with available sequences in GenBank using a BLAST search [36]. The strain exhibited 96% nucleotide sequence similarities with *Halopiger xanaduensis* [3]. These values were lower than the 98.7% 16S rRNA gene sequence threshold recommended by Stackebrandt and Ebers to delineate a new species without carrying out DNA-DNA hybridization [37]. A phylogenetic tree (Figure 1) was constructed using the neighbor-joining method with the MEGA 5 program package [38] after multiple alignments of the data using MUSCLE [39]. Evolutionary distances were calculated using the Tamura-Nei model [40].

Phenotypic characterization was carried out according to the recommended minimal standards for the description of new taxa in the order *Halobacteriales* [41]. Table 2 summarizes the differential phenotypic characteristics of *H. goleamassiliensis* sp. nov. IIH3^T^, *H. xanaduensis* SH-6T, *H. aswanensis* 56^T^ and *H. salifodinae* KCY076B2^T^. Different growth temperatures (30, 37, 40, 50, 55, 60°C), pH values (5, 6, 7, 7.5, 8, 8.5, 9, 10, 11, 12) and NaCl concentrations (0, 10, 12, 15, 20, 22.5, 25, 30% W/V) were tested on strain IIH3^T^. Cell growth was observed between 40°C and 60°C (optimum at 55°C), between 15% and 30% NaCl (optimum at 22.5-25 % NaCl) and at 7 to 11 pH values (optimum at pH 8).

**Table 2:** Differential phenotypic characteristics between strain IIH3^T^ and related species

**Characteristic**	***H. goleamassiliensis***	***H. xanaduensis***	***H. aswanensis***	***H. salifodinae***
Cell morphology	coccus	pleomorphic	pleomorphic	pleomorphic rods
Cell diameter (µm)	0.8-1.5	0.5-1.0×3.0-13.0	1.25-6.50×0.6–0.9	ND
Pigmentation	salmon	red	pink	cream
Oxygen requirement	strictly aerobic	strictly aerobic	strictly aerobic	strictly aerobic
Gram stain	negative	negative	negative	negative
NaCl range (%,w/v)	15-30	15-30	10- 30	11-31
NaCl optimum (%,w/v)	22.5-25	25	22.5-25	17-20
Temperature range (°C)	40-60	28-45	40-50	25-50
Temperature optimum (°C)	55	37	40	37-45
pH range	7-11	6 -11	6-9.2	6-8
pH optimum	8-8.5	7.5-8	7.5	7.0
Motility	non-motile	non-motile	motile	non-motile
Catalase	+	+	+	+
**hydrolysis of**				
Starch	-	-	+	-
Tween 80	+	+	+	-
Casein	-	-	-	ND
Gelatin	+	+	-	-
Lipids from egg yolk	+	ND	-	ND
**utilization of**				
D-Glucose	+	+	+	+
Galactose	+	+	ND	-
D-Xylose	+	+	+	-
Lactose	+	-	-	-
Fructose	-	-	+	-
Starch	-	-	+	+
Mannose	+	-	ND	+
D-Ribose	+	-	ND	-
Sucrose	-	ND	+	ND
Rhamnose	+	ND	ND	-
Mannitol	-	-	ND	ND
Citrate	-	-	ND	-
L-Arginine	-	-	-	-
Indole production	-	-	+	-
Urease	-	+	-	-
H2S production	-	-	+	+

Strains: *H. goleamassiliensis* sp. nov. IIH3^T^; *H. xanaduensis* SH-6T; 3, *H. aswanensis*; *H. salifodinae* KCY076B2^T^.

+: Positive result, -: Negative result, ND: Not Determined

**Figure 2:**
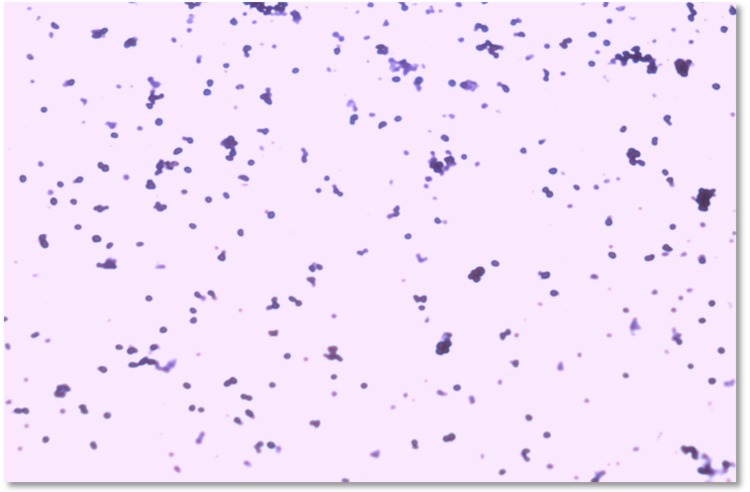
Gram staining of *Halopiger goleamassiliensis* strain IIH3^T^.

**Figure 3:**
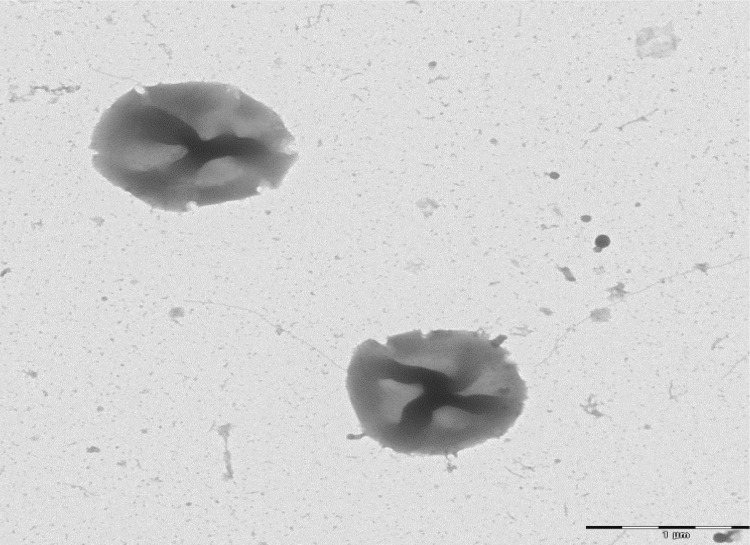
Transmission electron microscopy of *H. goleamassiliensis* strain IIH3^T^, using a Morgani 268D (Philips) at an operating voltage of 60kV. The scale bar represents 1µm.

Under optimal growth conditions on SG agar medium and after incubation for 15-20 days at 55°C, colonies were salmon pigmented, circular with a diameter of 1-2 mm. Cell morphology and motility were examined by using light microscopy and phase-contrast microscopy. Gram staining was performed using samples fixed with acetic acid, as described by Dussault in 1955 [42]. Cells are Gram-negative, cocci (Figure 2) measuring 0.8-1.5 μm in diameter (Figure 3). Motility and spores or capsules were not observed.

All the following biochemical and nutritional tests were realized in duplicate. Strain IIH3^T^ was found to be oxidase- and catalase- positive. The strain is extremely halophilic and cell lysis is observed in distilled water. It is a strictly aerobic organism and anaerobic growth does not occur even in the presence of KNO_3_ or arginine. Neither magnesium nor amino acids are required for growth. Tween 80, gelatin, and lipids from egg yolk are hydrolysed, whereas urea, starch, casein, and phosphatase are not. Production of indole and methyl red, Voges–Proskauer and Simmons' citrate tests are negative. H_2_S is not produced from cysteine.

Utilization of carbohydrates and other compounds as sole carbon sources and acid production from these compounds were determined as described by Oren [41]. Several sugars and amino acids can serve as sole carbon and energy sources (Table 2).

Antibiotic sensitivity tests were determined on SG medium agar plates with antibiotic discs. Strain IIH3^T^ is susceptible to bacitracin (10 μg), novobiocin (30 μg), streptomycin (10 μg) and sulfamethoxazole (25 μg), but resistant to ampicillin (10 μg), cephalothin (30 μg), chloramphenicol (30 μg), erythromycin (15 μg), gentamicin (10 μg), kanamycin (30 μg), nalidixic acid (30 μg), penicillin G (10 μg), rifampicin (30 μg), tetracycline (30 μg), and vancomycin (30 μg).

Matrix-assisted laser desorption/ionization time-of-flight mass spectrometry (MALDI-TOF MS is considered a reliable and rapid identification method for extremophilic prokaryotes [22,23] and it is used in the present study to characterize the strain IIH3^T^ as previously described [6-18]. A pipette tip was used to pick one isolated archaeal colony from a culture agar plate, and to spread it as a thin film on a MTP 384 MALDI-TOF tar-get plate (Bruker Daltonics, Leipzig, Germany). The colonies from strain IIH3^T^ and from other species of archaea were spotted in triplicate. After air-drying, 1.5 μl of matrix solution (a saturated solution of α-cyano-4-hydroxycinnaminic acid [CHCA] in 50% aqueous acetonitrile containing 2.5% trifluoroacetic acid) per spot was applied and allowed to dry for five minutes.

**Figure 4:**
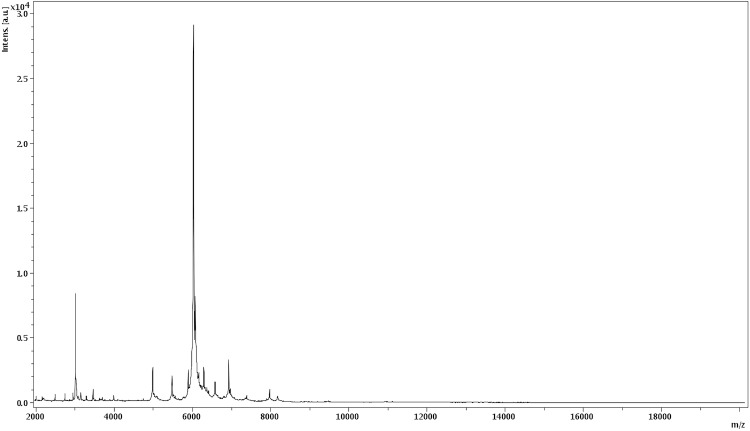
Reference mass spectrum from *H. goleamassiliensis* strain IIH3^T^. Spectra from 12 individual colonies were compared and a reference spectrum was generated.

**Figure 5:**
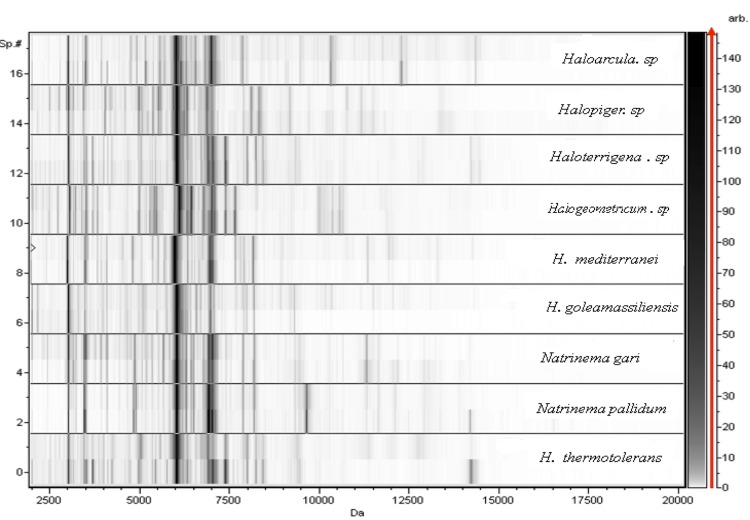
Gel view comparing the *H. goleamassiliensis* strain IIH3^T^ spectrum with those of other archaea. The Gel View displays the raw spectra of all loaded spectrum files arranged in a pseudo-gel like look. The x-axis records the m/z value. The left y-axis displays the running spectrum number originating from subsequent spectra loading. The peak intensity is expressed by the gray scale intensity. The scale shown on the right y-axis links the color to the peak intensity in arbitrary units.

Mass spectrometric measurements were performed with a Microflex spectrometer (Bruker). Spectra were recorded in the positive linear mode for the mass range of 2000 to 20,000 DA. The acceleration voltage was 20 kV. The time of acquisition was between 30 seconds and 1 minute per spot. Spectra were collected as a sum of 240 shots across a spot. Preprocessing and identification steps were performed using the manufacturer’s parameters. The IIH3^T^ spectrum (Figure 4) was imported into the MALDI BioTyper software (version 2.0, Bruker) and analyzed by standard pattern matching (with default parameter settings) against the spectra of *Haloferax mediterranei*, *Natrinema gari*, *Natrinema pallidum*, *Haloterrigena thermotolerans*, *Haloterrigena. sp*, *Halogeometricum. sp, Haloarcua. sp* and *Halopiger. sp* used as reference data in the BioTyper database (Figure 5).

A score enabled the identification, or not, from the tested species: a score > 2.3 with a validly published species enabled the identification at the species level, a score > 1.7 but < 2 enabled the identification at the genus level; and a score < 1.7 did not enable any identification. For strain IIH3^T^, none of the obtained scores was > 1, thus suggesting that our isolate was not a member of a known species. We added the spectrum from strain IIH3^T^ to our database for future reference. Figure 5 shows the MALDI-TOF MS spectrum differences between *H. goleamassiliensis* and other archaea.

## Genome sequencing information

### Genome project history

The organism was selected for sequencing on the basis of its phylogenetic position and 16S rRNA similarity to other members of the genus *Halopiger,* and as part of a study of archaeal diversity in hypersaline lakes of Algeria. It is the second genome of a *Halopiger* species and the first sequenced genome of *H. goleamassiliensis* sp. nov. The EMBL accession number is CBMB010000001-CBMB010000011 and it consists of 3 scaffolds (HG315690-HG315692). A summary of the project information (PRJEB1780) and its association with MIGS version 2.0 recommendations [27] is shown in the Table 3.

**Table 3:** Project information

**MIGS ID**	**Property**	**Term**
MIGS-31	Finishing quality	High-quality draft
MIGS-28	Libraries used	Paired-end 5 kb library
MIGS-29	Sequencing platforms	454 GS FLX Titanium
MIGS-31.2	Fold coverage	21.6×
MIGS-30	Assemblers	Newbler version 2.5.3
MIGS-32	Gene calling method	Prodigal
	EMBL ID	CBMB010000001- CBMB010000011
	EMBL Date of Release	June 18, 2018
	Project relevance	Study of the archaeal diversity in hypersaline lakes of Algeria

### Growth conditions and DNA isolation

*H. goleamassiliensis* sp.nov. strain IIH3^T^ (= CSUR P3036 =DSM on-going deposit) was grown in SG medium at 55°C in aerobic condition. DNA was isolated and purified using the Genomic DNA purification kit, NucleoSpin Tissue procedure (MACHEREY-NAGEL) following the standard protocol as recommended by the manufacturer. The quality of the DNA was checked on an agarose gel (0.8%) stained with SYBR safe. The yield and the concentration were measured by the Quant-it Picogreen Kit (Invitrogen) on the Genios Tecan Fluorometer at 33.1 ng/µL.

### Genome sequencing and assembly

A 5 kb paired-end sequencing strategy (Roche, Meylan, France) was used. This project was loaded on a 1/4 region on PTP Picotiterplate (Roche). Three µg of DNA was mechanically fragmented on the Covaris device (KBioScience-LGC Genomics, Teddington, UK) using miniTUBE-Red 5Kb. The DNA fragmentation was visualized through an Agilent 2100 BioAnalyzer on a DNA labchip 7500 with an optimal size of 4.7 kb. The library was constructed according to the 454 GS FLX Titanium paired end-protocol. After PCR amplification through 17 cycles followed by double size selection, the single stranded paired-end library was then loaded on a DNA labchip RNA pico 6000 on the BioAnalyzer. The pattern showed an optimum at 480 bp and the concentration was quantified on a Genios Tecan fluorometer at 642 pg/µL. The concentration equivalence of the library was calculated at 10^8^ molecules/µL. The library was stored at -20°C until further use, and amplified in 2 emPCR reactions at 0.25 cpb, in 2 emPCR at 0.5 cpb and in 2 emPCR at 1 cpb with the GS Titanium SV emPCR Kit (Lib-L) v2 (Roche). The yield of the 3 types of paired-end emPCR reactions was 3.68%, 8.05% and 10.69% respectively, in the quality range of 5 to 20% expected from the Roche procedure. These emPCR were pooled. Both libraries were loaded onto GS Titanium PicoTiterPlates (PTP Kit 70×75, Roche) and pyrosequenced with the GS Titanium Sequencing Kit XLR70 (Roche). The run was performed overnight and then analyzed on the cluster through the gsRunBrowser and Newbler assembler (Roche).

A total of 271,702 filter-passed wells were obtained and generated 84.39 Mb with an average length of 325 bp. The passed filter sequences were assembled using Newbler with 90% identity and 40 bp overlap. The final assembly contained 12 contigs (11 large contigs >1500 bp) arranged in 3 scaffolds and generated a genome size of 3.9 Mb, which corresponds to a coverage of 21.6× genome equivalent.

### Genome annotation

Open Reading Frames (ORFs) were predicted using prodigal with default parameters [43]. ORFs spanning a sequencing gap region were excluded. Assessment of protein function was obtained by comparing the predicted protein sequences with sequences in the GenBank [44] and the Clusters of Orthologous Groups (COG) databases using BLASTP. RNAmmer [45] and tRNAscan-SE 1.21 [46] were used for identifying the rRNAs and tRNAs, respectively. SignalP [47] and TMHMM [48] were used to predict signal peptides and transmembrane helices, respectively. For alignment lengths greater than 80 amino acids, ORFans were identified if their BLASTP E-value was lower than 1e-03. An E-value of 1e-05 was used if alignment lengths were smaller than 80 amino acids. DNA Plotter [49] was used for visualization of genomic features and Artemis [50] was used for data management. The mean level of nucleotide sequence similarity was estimated at the genome level between *H. goleamassiliensis* and 5 other members of the *Halobacteriaceae* family (Table 6), by BLASTN comparison of orthologous ORFs in pairwise genomes. Orthologous proteins were detected using the Proteinortho software using the following parameters: e-value 1e-05, 30% identity, 50% coverage and 50% of algebraic connectivity [51].

**Table 6:** Orthologous gene comparison and average nucleotide identity of *H. goleamassiliensis* with other compared genomes (upper right, numbers of orthologous genes; lower left, mean nucleotide identities of orthologous genes). Bold numbers indicate the numbers of genes or each genome.

Species (accession number)	*H. goleamassiliensis*	*N. pharaonis*	*H.* *turkmenica*	*N. magadii*	*H. jeotgali*	*H. xanaduensis*
*Halopiger goleamassiliensis* (PRJEB1780)	**3854**	1415	2036	1859	1542	2103
*Natronomonas pharaonis* (NC_007426)	67.60	**2659**	1393	1321	1254	1381
*Haloterrigena turkmenica* (NC_013743)	78.21	67.81	**3739**	1765	1559	2057
*Natrialba magadii* (NC_013922)	76.27	66.85	76.83	**3559**	1442	1828
*Halalkalicoccus jeotgali* (NC_014297)	68.70	67.76	68.97	67.55	**3035**	1589
*Halopiger xanaduensis* (NC_015666)	78.62	67.52	79.73	76.98	68.83	**3588**

## Genome properties

**Figure 6:**
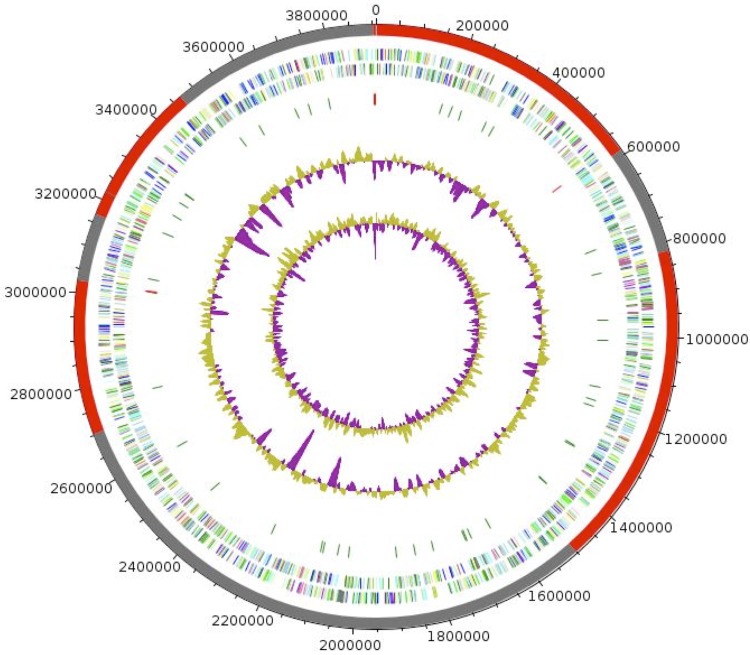
Graphical circular map of the *H. goleamassiliensis* IIH3^T^ genome. From the outside in: The first circle indicates the scaffolds, the next two circles show open reading frames oriented in the forward and reverse (colored by COG categories) directions, respectively. The fourth circle displays the rRNA gene operon (red) and tRNA genes (green). The fifth circle shows the G+C% content plot. The inner-most circle shows the GC skew, purple and olive indicating negative and positive values, respectively.

The genome is 3,906,923 bp long and displays a G+C content of 66.06%. (Table 4, Figure 6) It is composed of 12 contigs (11 large contigs >1,500 bp) arranged into 3 scaffolds. Of the 3,903 predicted genes, 3,854 were protein-coding genes (COG), and 49 were RNAs (1 gene is 16S rRNA, 1 gene is 23S rRNA, 3 genes are 5S rRNA, and 44 are tRNA genes). A total of 2,359 genes (61.21%) were assigned a putative function (by COG or by NR BLAST) and 188 genes were identified as ORFans (4.88%). The remaining genes were annotated as hypothetical proteins (1059 genes = 27.48%). The distribution of genes into COG functional categories is presented in Table 4. The properties and the statistics of the genome are summarized in Tables 4 and 5.

**Table 4:** Nucleotide content and gene count levels of the genome

**Attribute**	Value	% of total^a^
Genome size (bp)	3,906,923	100
DNA G+C content (bp)	2,581,064	66.06
DNA coding region (bp)	3,359,291	85.98
Total genes	3,903	100
RNA genes	49	1.26
Protein-coding genes	3,854	98.74
Genes with function prediction	2,359	61.21
Genes assigned to COGs	2,446	63.47
Genes with peptide signals	320	8.30
Genes with transmembrane helices	906	23.51

^a^ The total is based on either the size of the genome in base pairs or the total number of protein coding genes in the annotated genome.

**Table 5:** Number of genes associated with the 25 general COG functional categories

**Code**	**Value**	**% age^a^**	**Description**
J	166	4.31	Translation
A	1	0.003	RNA processing and modification
K	157	4.07	Transcription
L	113	2.93	Replication, recombination and repair
B	3	0.08	Chromatin structure and dynamics
D	18	0.47	Cell cycle control, mitosis and meiosis
Y	0	0	Nuclear structure
V	46	1.19	Defense mechanisms
T	128	3.32	Signal transduction mechanisms
M	74	1.92	Cell wall/membrane biogenesis
N	51	1.32	Cell motility
Z	0	0	Cytoskeleton
W	0	0	Extracellular structures
U	27	0.70	Intracellular trafficking and secretion
O	114	2.96	Post-translational modification, protein turnover, chaperones
C	168	4.36	Energy production and conversion
G	122	3.17	Carbohydrate transport and metabolism
E	266	6.90	Amino acid transport and metabolism
F	70	1.82	Nucleotide transport and metabolism
H	131	3.40	Coenzyme transport and metabolism
I	107	2.78	Lipid transport and metabolism
P	176	4.57	Inorganic ion transport and metabolism
Q	82	2.13	Secondary metabolites biosynthesis, transport and catabolism
R	510	13.23	General function prediction only
S	248	6.43	Function unknown
-	1408	36.53	Not in COGs

^a^ The total is based on the total number of protein coding genes in the annotated genome.

## Comparison with other genomes of archaea

Currently, only one genome from *Halopiger* species is available. Here, we compared the genome of *H. goleamassiliensis* strain IIH3^T^ with those of *H. xanaduensis* strain SH-6, *Halalkalicoccus jeotgali* strain B3, *Natronomonas pharaonis* strain DSM 2160, *Haloterrigena turkmenica* strain DSM 5511 and *Natrialba magadii* strain ATCC 43099. The genome of *H. goleamassiliensis* (3.90 Mb) is larger than that of *Halalkalicoccus jeotgali* and *Natronomonas pharaonis* (3.69 and 2.75 Mb, respectively) but of a smaller size than *H. xanaduensis*, *Natrialba magadii* and *Haloterrigena turkmenica* (4.35, 4.44 and 5.44 Mb respectively). The GC% content of *H. goleamassiliensis* (66.06%) is higher than that of *H. xanaduensis* (65.2%), *Haloterrigena turkmenica* (64.26%), *Natronomonas pharaonis* (63.1%), *Halalkalicoccus jeotgali* (62.5%) and *Natrialba magadii* (61.1%). *H. goleamassiliensis* has more predicted protein-coding genes (3,854) than *Haloterrigena turkmenica, H. xanaduensisNatrialba magadii, Halalkalicoccus jeotgali* and *Natronomonas pharaonis* (3,739, 3,588, 3,559, 3,035 and 2,659 respectively). In addition, *H. goleamasiliensis* shared a mean genomic sequence similarity of 67.60, 78.21, 76.27, 68.70 and 78.62% with *Natronomonas pharaonis*, *Haloterrigena turkmenica*, *Natrialba magadii*, *Halalkalicoccus jeotgali* and *Halopiger xanaduensis* respectively (Table 6).

## Conclusion

On the basis of phenotypic, phylogenetic and genomic analyses, we formally propose the creation of *Halopiger goleamassiliensis* sp. nov. that contains the strain IIH3^T^. This archaeal strain has been found in Algeria.

### Description of *Halopiger goleamassiliensis* sp. nov.

***Halopiger goleamassiliensis*** (go.le’a. ma. si. li. en’sis. L. gen. masc. n. goleamassiliensis from the combination of **El Golea**, the Algerian region where the strain was isolated, and massiliensis, of Massilia, the Latin name of Marseille where the strain was sequenced). It has been isolated from an evaporitic sediment in El Golea Lake, Algeria.

Colonies were smooth, salmon-pigmented and small with 1 to 2 mm in diameter under optimal growth conditions. Strain is strictly aerobic, extremely halophilic and moderately thermophilic archaeon. Growth occurs at NaCl concentrations of 15-30%, at pH values in the range 7-11, and within the temperature range 40-60 °C. Optimal NaCl concentration, pH and temperature for growth are 22.5-25%, 8.0 and 55 °C, respectively. Magnesium is not required for growth. Cells are coccus-shaped (0.8-1.5 µm), Gram-negative, non-motile and lyse in distilled water. Cells are positive for catalase, oxidase and lysine decarboxylase production and negative for urease, arginine dihydrolase, ornithine decarboxylase, tryptophanase, phosphatase, β-galactosidase, D-mannitol, sacharose, starch, dextrose, and D-fructose fermentation. The following substrates are utilized as single carbon and energy sources for growth: pyruvate, D-glucose, D-mannose, D-ribose, D-xylose, maltose, sucrose, lactose, casamino acids, bacto-peptone, bacto-tryptone, and yeast extract. Tween 80, gelatin, and lipids from egg yolk are hydrolysed, whereas urea, starch, and casein are not. Methyl red, Voges–Proskauer, Simmons' citrate tests, and H_2_S production are negative.

Cells are susceptible to bacitracin, novobiocin, streptomycin, and sulfamethoxazole but resistant to ampicillin, cephalothin, chloramphenicol, erythromycin, gentamicin, kanamycin, nalidixic acid, penicillin G, rifampicin, tetracycline, and vancomycin.

The G+C content of the DNA is 66.06%. The 16S rRNA and genome sequences are deposited in GenBank and EMBL under accession numbers KC430940 and CBMB010000001-CBMB010000011, respectively. The type strain IIH3^T^ (=CSUR P3036 = DSM on-going deposit) was isolated from an evaporitic sediment in El Golea Lake, Algeria.

## References

[1] Ma Y Galinski EA Grant WD Oren A Ventosa A Halophiles 2010: life in saline environments. Appl Environ Microbiol 2010; 76:6971-6981 10.1128/AEM.01868-10 20817804 PMC2976238

[2] Euzéby JP. List of prokaryotic names with standing in nomenclature LPSN. 2011; Available at:http://www.bacterio.cict.fr/classifgenerafamilies.html#Halobacteriaceae Accessed 23 June 2013.

[3] Gutiérrez MC Castillo AM Kamekura M Xue Y Ma Y Cowan DA Jones BE Grant WD Ventosa A *Halopiger xanaduensis* gen. nov., sp. nov., an extremely halophilic archaeon isolated from saline Lake Shangmatala in Inner Mongolia, China. Int J Syst Evol Microbiol 2007; 57:1402-1407 10.1099/ijs.0.65001-0 17625165

[4] Hezayen FF Gutiérrez MC Steinbüchel A Tindall BJ Rehm BH *Halopiger aswanensis* sp. nov., a polymer-producing and extremely halophilic archaeon isolated from hypersaline soil. Int J Syst Evol Microbiol 2010; 60:633-637 10.1099/ijs.0.013078-0 19654343

[5] Zhang WY Jr Meng Y Zhu XF Wu M *Halopiger salifodinae* sp. nov., an extremely halophilic archaeon isolated from a salt mine. Int J Syst Evol Microbiol 2013 23563233 10.1099/ijs.0.050971-0

[6] Lagier JC El Karkouri K Nguyen TT Armougom F Raoult D Fournier PE Non-contiguous finished genome sequence and description of *Anaerococcus senegalensis* sp. nov. Stand Genomic Sci 2012; 6:116-125 10.4056/sigs.2415480 22675604 PMC3359877

[7] Lagier JC Gimenez G Robert C Raoult D Fournier PE Non-contiguous finished genome sequence and description of *Herbaspirillum massiliense* sp. nov. Stand Genomic Sci 2012; 7:200-209 23407294 10.4056/sigs.3086474 PMC3569391

[8] Hugon P Mishra AK Lagier JC Nguyen TT Couderc C Raoult D Fournier PE Non contiguous finished genome sequence and description of *Brevibacillus massiliensis* sp. nov. Stand Genomic Sci 2013; 8:1-14 10.4056/sigs.3466975 23961307 PMC3739172

[9] Hugon P Mishra AK Robert C Raoult D Fournier PE Non-contiguous finished genome sequence and description of *Anaerococcus vaginalis.* Stand Genomic Sci 2012; 6:356-365 10.4056/sigs.2716452 23407456 PMC3558966

[10] Mishra AK Hugon P Robert C Raoult D Fournier PE Non contiguous-finished genome sequence and description of *Peptoniphilus grossensis* sp. nov. Stand Genomic Sci 2012; 7:320-330 23408485 10.4056/sigs.3076460 PMC3569384

[11] Kokcha S Mishra AK Lagier JC Million M Leroy Q Raoult D Fournier PE Non contiguous-finished genome sequence and description *of Bacillus timonensis* sp. nov. Stand Genomic Sci 2012; 6:346-355 10.4056/sigs.2776064 23408487 PMC3558959

[12] Lagier JC Armougom F Mishra AK Nguyen TT Raoult D Fournier PE Non-contiguous finished genome sequence and description of *Alistipes timonensis* sp. nov. Stand Genomic Sci 2012; 6:315-324 23408657 10.4056/sigs.2685971 PMC3558960

[13] Ramasamy D Kokcha S Lagier JC Nguyen TT Raoult D Fournier PE Genome sequence and description of *Aeromicrobium massiliense* sp. nov. Stand Genomic Sci 2012; 7:246-257 10.4056/sigs.3306717 23408663 PMC3569385

[14] Mishra AK Lagier JC Robert C Raoult D Fournier PE Non-contiguous finished genome sequence and description of *Clostridium senegalense* sp. nov. Stand Genomic Sci 2012; 6:386-395 23408737 10.4056/sigs.2766062 PMC3558962

[15] Lagier JC Ramasamy D Rivet R Raoult D Fournier PE Non contiguous-finished genome sequence and description of *Cellulomonas massiliensis* sp. nov. Stand Genomic Sci 2012; 7:258-270 10.4056/sigs.3316719 23408774 PMC3569388

[16] Kokcha S Ramasamy D Lagier JC Robert C Raoult D Fournier PE Non-contiguous finished genome sequence and description of *Brevibacterium senegalense* sp. nov. Stand Genomic Sci 2012; 7:233-245 10.4056/sigs.3256677 23408786 PMC3569389

[17] Mishra AK Lagier JC Robert C Raoult D Fournier PE Non contiguous-finished genome sequence and description of *Peptoniphilus timonensis* sp. nov. Stand Genomic Sci 2012; 7:1-11 10.4056/sigs.2956294 23449949 PMC3570796

[18] Mishra AK Lagier JC Rivet R Raoult D Fournier PE Non-contiguous finished genome sequence and description of *Paenibacillus senegalensis* sp. nov. Stand Genomic Sci 2012; 7:70-81 23459006 10.4056/sigs.3056450 PMC3577113

[19] Tindall BJ Rosselló-Móra R Busse HJ Ludwig W Kämpfer P Notes on the characterization of prokaryote strains for taxonomic purposes. Int J Syst Evol Microbiol 2010; 60:249-266 10.1099/ijs.0.016949-0 19700448

[20] Klenk HP Göker M En route to a genome-based classification of Archaea and Bacteria. Syst Appl Microbiol 2010; 33:175-182 10.1016/j.syapm.2010.03.003 20409658

[21] Schleifer KH Classification of Bacteria and Archaea: past, present and future. Syst Appl Microbiol 2009; 32:533-542 10.1016/j.syapm.2009.09.002 19819658

[22] Dridi B Raoult D Drancourt M Matrix-assisted laser desorption/ionization time-of-flight mass spectrometry identification of Archaea: towards the universal identification of living organisms. APMIS 2012; 120:85-91 10.1111/j.1600-0463.2011.02833.x 22229263

[23] Krader P Emerson D Identification of archaea and some extremophilic bacteria using matrix-assisted laser desorption/ionization time-of-flight (MALDI-TOF) mass spectrometry. Extremophiles 2004; 8:259-268 10.1007/s00792-004-0382-7 15042434

[24] Ozcan B, Cokmus C, Coleri A, Caliskan M. Characterization of extremely halophilic archaea isolated from saline environment in different parts of Turkey. Mikrobiologiia. 2006; **75**(6): 849-856. 17205811

[25] Field D Garrity G Gray T Morrison N Selengut J Sterk P Tatusova T Thomson N Allen MJ Angiuoli SV The minimum information about a genome sequence (MIGS) specification. Nat Biotechnol 2008; 26:541-547; http://www.ncbi.nlm.nih.gov/entrez/query.fcgi?cmd=Retrieve&db=PubMed&list_uids=18464787&dopt=Abstract 10.1038/nbt1360 18464787 PMC2409278

[26] Woese CR Kandler O Wheelis ML Towards a natural system of organisms: proposal for the domains Archaea, Bacteria, and Eucarya. Proc Natl Acad Sci USA 1990; 87:4576-4579 10.1073/pnas.87.12.4576 2112744 PMC54159

[27] Garrity GM, Holt JG. Phylum AII. Euryarchaeota phy. nov. In: Garrity GM, Boone DR, Castenholz RW (eds), Bergey's Manual of Systematic Bacteriology, Second Edition, Volume 1, Springer, New York, 2001, p. 211-355.

[28] List Editor Validation List no. 85. Validation of publication of new names and new combinations previously effectively published outside the IJSEM. Int J Syst Evol Microbiol 2002; 52:685-690 10.1099/ijs.0.02358-0 12054225

[29] Grant WD, Kamekura M, McGenity TJ, Ventosa A. Class III. Halobacteria class. nov. In: Garrity GM, Boone DR, Castenholz RW (eds), Bergey's Manual of Systematic Bacteriology, Second Edition, Volume 1, Springer, New York, 2001, p. 294.

[30] Grant WD, Larsen H. Group III. Extremely halophilic archaeobacteria. Order Halobacteriales ord. nov. In Holt JG (ed), Bergey's Manual of Systematic Bacteriology, Volume 3, Baltimore : Williams & Wilkins, 1989, p. 2216-2228.

[31] The nomenclatural types of the orders *Acholeplasmatales*, *Halanaerobiales*, *Halobacteriales*, *Methanobacteriales*, *Methanococcales*, *Methanomicrobiales*, *Planctomycetales*, *Prochlorales*, *Sulfolobales*, *Thermococcales*, *Thermoproteales* and *Verrucomicrobiales* are the genera *Acholeplasma*, *Halanaerobium*, *Halobacterium*, *Methanobacterium*, *Methanococcus*, *Methanomicrobium*, *Planctomyces*, *Prochloron*, *Sulfolobus*, *Thermococcus*, *Thermoproteus* and *Verrucomicrobium*, respectively. Opinion 79. Int J Syst Evol Microbiol 2005; 55:517-518 10.1099/ijs.0.63548-0 15653928

[32] List Editor Validation List no. 31. Validation of the publication of new names and new combinations previously effectively published outside the IJSB. Int J Syst Bacteriol 1989; 39:495-497 10.1099/00207713-39-4-495

[33] Skerman VBD McGowan V Sneath PHA Approved Lists of Bacterial Names. Int J Syst Bacteriol 1980; 30:225-420 10.1099/00207713-30-1-225 20806452

[34] Gibbons NE. Family V. Halobacteriaceae Fam. nov. In: Buchanan RE, Gibbons NE (eds), Bergey's Manual of Determinative Bacteriology, Eighth Edition, The Williams and Wilkins Co., Baltimore, 1974, p. 269-273.

[35] Ashburner M Ball CA Blake JA Botstein D Butler H Cherry JM Davis AP Dolinski K Dwight SS Eppig JT Gene ontology: tool for the unification of biology. The Gene Ontology Consortium. Nat Genet 2000; 25:25-29 10.1038/75556 10802651 PMC3037419

[36] Altschul SF Madden TL Schaffer AA Zhang J Zhang Z Miller W Lipman DJ Gapped BLAST and PSI-BLAST: a new generation of protein database search programs. Nucleic Acids Res 1997; 25:3389-3402 10.1093/nar/25.17.3389 9254694 PMC146917

[37] Stackebrandt E, Ebers J. Taxonomic parameters revisited: tarnished gold standards. Microbiol Today. 2006; 152-155.

[38] Tamura K Peterson D Peterson N Stecher G Nei M Kumar S MEGA5: molecular evolutionary genetics analysis using maximum likelihood, evolutionary distance, and maximum parsimony methods. Mol Biol Evol 2011; 28:2731-2739 10.1093/molbev/msr121 21546353 PMC3203626

[39] Edgar RC MUSCLE: a multiple sequence alignment method with reduced time and space complexity. BMC Bioinformatics 2004; 5:113 10.1186/1471-2105-5-113 15318951 PMC517706

[40] Tamura K Nei M Estimation of the number of nucleotide substitutions in the control region of mitochondrial DNA in humans and chimpanzees. Mol Biol Evol 1993; 10:512-526 8336541 10.1093/oxfordjournals.molbev.a040023

[41] Oren A Ventosa A Grant WD Proposed minimal standards for description of new taxa in the order *Halobacteriales*. Int J Syst Bacteriol 1997; 47:233-238 10.1099/00207713-47-1-233

[42] Dussault HP An improved technique for staining red halophilic bacteria. J Bacteriol 1955; 70:484-485 13263323 10.1128/jb.70.4.484-485.1955 PMC386254

[43] Hyatt D Chen GL Locascio PF Land ML Larimer FW Hauser LJ Prodigal: prokaryotic gene recognition and translation initiation site identification. BMC Bioinformatics 2010; 11:119 10.1186/1471-2105-11-119 20211023 PMC2848648

[44] Benson DA Karsch-Mizrachi I Clark K Lipman DJ Ostell J Sayers EW GenBank. Nucleic Acids Res 2012; 40:D48-53 10.1093/nar/gkr1202 22144687 PMC3245039

[45] Lagesen K Hallin P Rodland EA Staerfeldt HH Rognes T Ussery DW RNAmmer: consistent and rapid annotation of ribosomal RNA genes. Nucleic Acids Res 2007; 35:3100-3108 10.1093/nar/gkm160 17452365 PMC1888812

[46] Lowe TM Eddy SR tRNAscan-SE: a program for improved detection of transfer RNA genes in genomic sequence. Nucleic Acids Res 1997; 25:955-964 9023104 10.1093/nar/25.5.955 PMC146525

[47] Bendtsen JD Nielsen H von Hiejne G Brunak S Improved prediction of signal peptides: SignalP 3.0. J Mol Biol 2004; 340:783-795 10.1016/j.jmb.2004.05.028 15223320

[48] Krogh A Larsson B von Heijne G Sonnhammer EL Predicting transmembrane protein topology with a hidden Markov model: application to complete genomes. J Mol Biol 2001; 305:567-580 10.1006/jmbi.2000.4315 11152613

[49] Carver T Thomson N Bleasby A Berriman M Parkhill J DNAPlotter: circular and linear interactive genome visualization. Bioinformatics 2009; 25:119-120 10.1093/bioinformatics/btn578 18990721 PMC2612626

[50] Rutherford K Parkhill J Crook J Horsnell T Rice P Rajandream MA Barrell B Artemis: sequence visualization and annotation. Bioinformatics 2000; 16:944-945 10.1093/bioinformatics/16.10.944 11120685

[51] Lechner M Findeiss S Steiner L Marz M Stadler PF Prohaska SJ Proteinortho: detection of (co-)orthologs in large-scale analysis. BMC Bioinformatics 2011; 12:124 10.1186/1471-2105-12-124 21526987 PMC3114741

[52] Buchanan RE, Gibbons NE. Family V. Halobacteriaceae fam. nov. In: Bergey's Manual of Systematic Bacteriology. 1974; p. 279.

